# Time-series analysis of trends in the incidence rates of successful and attempted suicides in Thailand in 2013–2019 and their predictors

**DOI:** 10.1186/s12888-022-04125-5

**Published:** 2022-08-31

**Authors:** Suwanna Arunpongpaisal, Sawitri Assanagkornchai, Virasakdi Chongsuvivatwong, Nattakorn Jampathong

**Affiliations:** 1 Epidemiology Department, Faculty of Medicine, Prince of Songkla University, 15 Karnjanavanich Rd., Hat Yai, Songkhla, 90110 Thailand; 2 KhonKaen Rajanagarindra Psychiatric Hospital, Khon Kaen, Thailand

**Keywords:** Suicide, Attempted suicide, Predictors, Trends, Thailand

## Abstract

**Background:**

Suicide rates are of increasing concern worldwide. There are approximately 4000–5000 deaths by suicide each year in Thailand. This study examined trends in annual incidence rates and predictors of successful and attempted suicides in Thailand (2013–2019).

**Methods:**

Secondary data analysis was conducted on data from two national-level databases: The National Health Security Office and the National Death Certification Registry System. Time-related trends and predictors of successful and attempted suicides were calculated using joinpoint regression and multivariable logistic regression analyses, respectively.

**Results:**

Of all successful suicide cases from 2013 to 2019, about 80% involved men, with an average age of 45.37 (± 16.43) years. Predictors of successful suicide included male sex, older age, using highly lethal methods, and no prior psychiatric treatment. Among individuals admitted to hospitals following a suicide attempt from 2013– to 2019, the average age at first admission was 38.83 ± 22.47 years, with women more heavily represented than men. Only 2.3% of these patients received psychiatric treatment in the hospital. Predictors of attempted suicide included female sex; adolescent or adult; and mental, alcohol, or substance-related disorder(s). Age-standardized annual rates per 100,000 people showed that, through 2019, suicide incidence increased slightly, and attempts decreased.

**Conclusions:**

There was a significantly increasing trend in successful suicide during the 7 years; the increase was more notable among men. The study highlights sex-related gaps in public health owing to an identified higher incidence of suicide among men, and a higher incidence of suicide attempts in women adolescents, emphasizing the need to consider sex-sensitive issues in individual as well as societal contexts.

## Background

Suicide is a complex phenomenon and a serious public health problem. Suicide rates are of increasing concern worldwide, with approximately 800,000 deaths annually. Nearly one-third of all suicides occur among young people. The global age-standardized suicide rate was 9.0 per 100,000 people in 2019—12.6 and 5.4 per 100,000 for men and women, respectively (2.3 times higher in men than women). Most successful suicides occurred in low- and middle-income countries (77%). For both sexes, suicide is the fourth leading cause of death after road injury, tuberculosis, and interpersonal violence in young people aged 15–29 years, and the third and fourth leading cause of death for women and men, respectively [1].

Thailand’s population was approximately 66.56 million in 2019, with 50.2% people living in urban areas and an overall poverty rate of 6.2% [2, 3]. There are approximately 4000–5000 deaths by suicide each year in Thailand. The official suicide rate was 6.64 per 100,000 people in 2019, with 76.5% aged 20–59 years, 21% aged 60 years and older, and 2.5% aged 10–19 years. Men/boys were 4.7 times more likely to complete suicide than women/girls. A high suicide rate was observed in the northern region of Thailand, with an average of 13.9 per 100,000 people from 1998 to 2002 [4]. However, the Thai official suicide rate was lower than what was estimated by the World Health Organization (WHO) (6.4 vs. 8.8, respectively, in 2019) [1, 5]. This may be owing to different methods used in the adjustment for underreporting and misclassification, which often occurs when suicide deaths are coded as “injury of undetermined intent” or “accidents” to avoid social stigma and a sense of disgrace among families [6]. It is well documented that Thailand has substantially reduced its poverty level and has achieved considerable gains in the health status of its overall population over recent decades; however, poor health conditions attributable to poverty remain a key problem. Approximately 55% of the Thai population resides in relatively disadvantaged areas located in the central, northeastern, and southern regions, with high mortality rates owing to liver cancer, diabetes, renal diseases, and suicide observed in these areas [7].

Studies on suicide etiology report the existence of multiple risk factors, including male sex, older age, social isolation, financial difficulties, unemployment, interpersonal hardships, and previous suicide attempts (SAS). Additionally, psychiatric disorders (including depression, bipolar disorder, schizophrenia, alcohol and substance abuse or dependence, and severe personality disorders), chronic pain, serious physical illness, and low accessibility to the health care system are common risk factors, whereas stressful life events often trigger suicidal intentions among those prone to experience them [8, 9]. Suicide attempts (SAS) refers to intentional self-inflicted poisoning, injury, or self-harm that may have a fatal intent or outcome [10]. The strongest predictors of suicide are previous SAS or having engaged in deliberate self-harm (with or without suicidal intent), which are found in 40–60% of successful suicides [11–13]. However, the number of SAS are often subject to undercounting, underreporting, and denial owing to political, cultural, and social reasons, as well as taboos existing for individuals and societies at large. The more influential these issues are, the more prevalent the underreporting and undercounting of SAS [14]. Nock MK et al, had reported the estimated lifetime prevalence of suicide attempts in the overall cross-national sample of 84,850 subjects in the 17 countries classified as developing countries (including China, Columbia, Lebanon, Mexico, Nigeria, South Africa, and Ukraine) and developed countries (including United States, Japan, New Zealand, Belgium, France, Germany, Italy, Netherlands, Spain, and Israel). Prevalence of SAS in developing countries ranged 0.7% to 4.7%, while in developing countries ranged 0.5% to 5.0% [15].

Suicide prevention is central to mental health policy in many countries, including Thailand. To date, no studies of national trends in the incidence of completed suicides or SAS have been conducted using Thailand’s mortality and national health data. The past decade has seen several social crises in Thailand, for example, a political crisis in 2013, reduced economic growth in 2015 following the Asian financial crisis, national grivance owing to the death of King Rama 9th in 2016, a flooding diaster in 2017, and recurring protests against the military government starting in 2018 [16]. These social stresses could precipitate or induce psychological distress among the individual population of the country, in turn influencing suicide or suicide attempt trends in this particular period. Examining suicide and suicide attempt trends in this period can provide some insight into how individuals respond to social situations. Under these circumstances, contexts may need to focus on immediate and feasible priorities for suicide prevention. The knowledge gaps are the trend of annual age-standardized suicide and suicide attempts rate in Thailand during several social crises and their predictors. Therefore, we analyzed the trends of the annual incidence of both successful suicides and SAS from 2013 to 2019 in Thailand to identify predictors of these variables. We hypothesize that trend of the annual incidence of successful suicide and SAS would show an increasing pattern during several social crises, and their predictors would be sex, age, methods of suicide, mental disorders, alcohol or substance use disorders, and disability. Acknowledging suicide and SAS trends is important to provide a more complete picture of the scale and nature of the problem, and in facilitating better-informed decisions concerning strategies around suicide prevention, such as targeting high-risk age/sex groups and focusing on more popular suicide methods that are potentially amenable to method-restriction policies. 

## Methods/design

### Study design and data sources

This study was a secondary data analysis using a time-series method. Two databases of Thai cases aged 10 years or older, recorded between January 1, 2013, and December 31, 2019, were used.

The first database contains mortality data from the National Death Certification Registry System, regularly maintained by the Bureau of Policy and Strategy of the Ministry of Public Health. In this database, there were 31,201 records of individuals with identified causes of death as intentional self-harm at the time of this study (ICD10 CM codes: X60-X84) [17]. These were included in the final analysis.

The second database was from the National Health Security Office (NHSO), including countrywide data of all inpatients registered under the universal health coverage system. The universal health coverage system covers medical insurance for 99.8% of the Thai population [18]. Each individual has a unique identifier, allowing an individual’s multiple attempts to be linked within their index case. The first inpatient admission between 2013 and 2019 was designated as the index admission. Of the total 780,567 inpatient records, 267,214 duplicated cases were excluded. Of all included cases (*N* = 513,353), 26,178 patients were admitted owing to intentional self-harm (ICD10 CM codes: X60-X84).

The two datasets could not be linked because case identification numbers were unavailable for security reasons. Thus, they were analyzed separately. The mid-year population in 2013–2019 was retrieved from the Strategy and Planning Division, Ministry of Public Health (https://bps.moph.go.th/new_bps/).

### Variables examined

The following variables were obtained from the mortality database: sex, age, place of birth, place of death, date of death, and causes of death with ICD10 CM codes: X60–X84. From the NHSO database, the following variables were obtained for each inpatient: date of birth, sex, place of birth, place of hospital admission, date of admission, primary and secondary diagnoses, self-harm methods used, date of discharge, discharge status, date of death, presence of disability, and psychiatric treatment obtained during admission. Self-harm or suicide methods were classified into high and low lethality based on the suicide acts resulting in death or hospitalization. A systematic review study recruited 10 708 articles to be screened and only 34 of them performed a meta-analysis for case fatality rate (CFR). High lethality methods included firearm (CFR 89.7%), hanging (CFR 84.5%), drowning (CFR 80.4%), gas poisoning (CFR 56.6%), and jumping (CFR 46.7%). Low lethality methods included drug/liquid poisoning (CFR 8%), cutting (CFR 4%) [19]. So, in this study, high lethality methods referred to firearm, hanging, drowning, jumping, gas poisoning, and car crash while low lethality methods referred to drug/chemical liquid poisoning, pesticide, herbicide, alcohol/organic solvent toxicity, cutting/ blunt injury, and other/unspecified means.

### Statistical analysis

The pooled incidence rates of completed suicides and SAS per 100,000 people per year over the study period, from 2013 to 2019, as well as annual rates per 100,000 people, were calculated based on the number of reported deaths and inpatients with code X60–X84 annually. Age- and sex-standardized suicide and SAS rates were then calculated. Annual suicide and SAS rate trends from 2009 to 2017 were then analyzed using the joinpoint regression program version 4.9.9.0, March 2021 [20]. The method of joinpoint was useful to assess changes in time series data and accurately identify if the population structure changes based on time series of abundance, as well as identify when this change occurs. The advantage of using joinpoint regression over regression methods is the constrain of continuity at the change-point(s) and the choice of the number of joinpoint(s) and their locations is estimated within the model which the minimum and the maximum number of joinpoints allowed arbitrarily set before the analysis while the final number of joinpoint(s) is not fixed a priori by the researcher, as in a classical piecewise regression model, but it is established on the basis of a statistical criterion [21]. Process of joinpoint [22] was conducted into 4 steps as the following: 1) creating input data files (suicide, and SAS excel type) with the variable columns: sex=by varable, year = independent variable, age group = adjusted variable, dependent variable =age-adjusted suicide rate that calculated by the number of suicide deaths in each sex, age groups and years divided by the mid year Thai population in each sex, age groups, years time with world standard population usingSEER*stat table accessed at website: https://seer.cancer.gov/stdpopulations/world.who.html 2) setting parameters - independent variables=year, dependent variable=age-adjusted suicide rate, adjusted variable=age group, by variable=sex, interval type=annual, choose an error model to fit= uncorrelated, set log transformation, then specified modelling method that constrained on the number of joinpoint =2, permutation test method with significant level 0.05 and number of permutation =4499, specified AAPC segmment range entire 2013 to 2019, and APC/AAPC/Tau confidence intervals as the parametric test, no advanced analysis. 3) executing the joinpoint regression program, joinpoint calculation engine processed the data and generated the output window to displayed the results. 4) viewing the joinpoint results on the graph, data, model estimates, trends, and model selection. Then SAS data was processed in the same 4 steps.

To examine predictors of SAS from the NHSO data (*N* = 513,353), we first performed a univariate analysis between SAS (dependence variable) and each independent variables, including sex; age group; self-harm method; having a disability; and presence of mental, alcohol-related, or substance-related disorder(s). All significant independent variables were entered into the multivariable logistic regression model. Finally, backward stepwise elimination was used to retain variables significantly associated with SAS.

Next, we conducted a subgroup analysis involving cases of SAS  from NHSO database (*n* = 26,178) to identify death predictors (i.e., successful suicide) following these SAS. Univariate and multivariablelogistic regression models were constructed in the manner described above. R program version 4.1.1 (2021-08-10) was used for analyses.

## Results

### Characteristics of successful and attempted suicides in 2013–2019

Of all successful suicide cases from 2013–to 2019, about 80% involved men, with an average age of 45.37 years (± 16.43) (44.5 ± 16.22 years in men and 48.8 ± 16.79 years in women). Approximately three-quarters (77.3%) of included cases were those who used highly lethal methods to kill themselves: hanging was the most common (73.4%), and others included gas poisoning, smoke inhalation via a fire/flame, drowning, gunshot, jumping from a high structure, and car crash. Of the less lethal methods, pesticide poisoning (16.41%) was the most commonly used, followed by chemical and drug poisoning. Men were significantly more likely to use high-lethality methods to commit suicide than women.

Among individuals admitted to hospitals following an SAS in 2013–2019, the average age at first admission was 38.83 ± 22.47 years, with women more heavily represented than men. In contrast to successful suicide cases, most (93.2%) involved less-lethal methods for SAS. Table 1 shows drug poisoning was the most frequently used method (45.34%), followed by pesticide poisoning (26.55%). The average length of hospital admission following each patient’s index admission was 4 hours (range: 0–24 hours). Only 2.3% of patients received psychiatric treatment in the hospital. Of these, 1% had at least one physical disability, whereas 37.8, 2.9, and 0.9% had mental, alcohol-related, and substance-related disorders, respectively. Moreover, 6.7% died during their hospitalization, with 11.8% dying after discharge. Most deaths after SAS (accounting for 64.5% of deaths) were men, with pesticide ingestion (accounting for 55.1% of deaths) being the most commonly used method (Table 1).

**Table 1:** Comparison of methods used by succesful suicides and inpatients  with attempted suicide based on 2013–2019 data

Method	Suicide deaths (*n* = 31,201)			Suicide attempts (*n* = 26,178)		
	Men, *n* = 24,867	Women, *n* = 6334	*p*	Men, *n* = 11,599	Women, *n* = 14,579	*p*
	n (%)	n (%)		n (%)	n (%)	
High-lethality methods	19,900 (80.0)	4207 (66.4)	< .001	1201 (10.4)	583 (4.0)	< .001
Hanging (X70)	18,786 (75.5)	4120 (65.0)	< .001	1055 (9.1)	469 (3.22)	< .001
Gas poisoning (X67)	20 (0.08)	12 (0.19)	.028	18 (0.16)	18 (0.12)	.491
Smoke fire-frame (X76–77)	39 (0.16)	6 (0.09)	.328	60 (0.52)	26 (0.18)	< .001
Drowning (X71)	31 (0.12)	15 (0.24)	.058	24 (0.21)	43 (0.29)	0161
Gunshot (X72–75)	952 (3.83)	37 (0.58)	< .001	22 (0.19)	5 (0.03)	0.0001
Jumping from a high place (X80–81)	63 (0.25)	17 (0.27)	.942	22 (0.19)	20 (0.14)	.292
Car crash (X82)	9 (0.04)	0 (0)	N/A	0 (0)	2 (0.01)	N/A
Low-lethality methods	4967 (20.0)	2127 (33.6)	< .001	10,398 (89.6)	13,996 (96.0)	< .001
Drug overdose (X60–64)	270 (1.09)	169 (2.67)	< .001	3480 (30.0)	8395 (57.58)	< .001
Pesticide (X68)	3660 (14.72)	1461 (23.07)	< .001	4424 (38.14)	2529 (17.35)	< .001
Chemical poison (X69)	600 (2.41)	414 (6.54)	< .001	2097 (18.08)	2795 (19.17)	.024
Alcohol toxicity (X65–66)	10 (0.04)	1 (0.02)	.583	184 (1.59)	140 (0.96)	< .001
Knife/blunt instrument (X78–79)	108 (0.43)	13 (0.21)	.012	107 (0.92)	67 (0.46)	< .001
Other means (X83–84)	319 (0.56)	69 (1.09)	.239	106 (0.91)	70 (0.48)	< .001

### Annual age-standardized successful and attempted suicide rates from 2013 to 2019

The highest age-standardized suicide incidence rate was 8.95 per 100,000 people (14.8 in men and 3.4 in women), occurring in 2019. This year also recorded the highest rate of suicides among working-age adults (25–64 years). In subsequent years, the suicide rate among young men (15–24 years) increased (from 1.0 to 1.2 per 100,000 people). Additionally, the annual age-standardized incidence rates among women were consistently 3–4 times lower than those among men across this entire period (Table 2A).

**Table 2:** Age-standardized successful and attempted suicide rate in Thailand from 2013 to 2019

A. Age-standardized successful suicide rate														
Year	2013		2014		2015		2016		2017		2018		2019	
Sex	M	W	M	W	M	W	M	W	M	W	M	W	M	W
Age group (years)														
10–14	0.1	0.0	0.0	0.0	0.0	0.0	0.0	0.0	0.0	0.0	0.0	0.0	0.0	0.0
15–24	1.0	0.3	1.1	0.1	1.0	0.2	1.0	0.2	1.1	0.2	1.2	0.2	1.2	0.2
25–34	2.2	0.4	2.1	0.4	2.1	0.3	2.0	0.4	2.2	0.3	2.5	0.4	3.1	0.4
35–44	2.4	0.6	2.2	0.5	2.6	0.5	2.5	0.5	2.5	0.5	2.6	0.5	3.5	0.7
45–54	1.7	0.6	1.8	0.6	2.1	0.6	1.9	0.7	2.1	0.7	2.2	0.6	2.8	0.7
55–64	1.2	0.4	1.2	0.5	1.4	0.5	1.4	0.5	1.6	0.4	1.6	0.6	2.2	0.7
65–74	0.7	0.2	0.7	0.3	0.9	0.3	0.9	0.2	0.8	0.3	1.1	0.3	1.2	0.4
75–84	0.4	0.1	0.4	0.2	0.4	0.1	0.4	0.2	0.4	0.1	0.5	0.2	0.6	0.2
85–94	0.1	0.0	0.0	0.0	0.1	0.1	0.1	0.0	0.1	0.0	0.1	0.0	0.1	0.0
≥ 95	0.0	0.0	0.0	0.0	0.0	0.0	0.0	0.0	0.0	0.0	0.0	0.0	0.0	0.0
Total	9.8	2.6	9.6	2.7	10.5	2.5	10.3	2.6	10.9	2.6	11.9	2.8	14.8	3.4
	6.12		6.08		6.46		6.35		6.63		7.27		8.95	
B. Age-standardized suicide attempt rate														
Year	2013		2014		2015		2016		2017		2018		2019	
Sex	M	W	M	W	M	W	M	W	M	W	M	W	M	W
Age group (years)														
10–14	0.1	0.3	0.2	0.6	0.2	0.5	0.2	0.7	0.1	0.8	0.2	0.5	0.1	0.3
15–24	1.5	3.0	2.2	4.4	1.1	2.5	1.2	2.5	0.9	2.1	1.0	2.1	0.7	1.5
25–34	0.9	0.5	1.0	0.5	0.8	0.5	0.7	0.4	0.6	0.3	0.7	0.4	0.7	0.6
35–44	0.6	0.7	0.9	0.7	0.6	0.6	0.6	0.5	0.7	0.5	0.7	0.5	0.6	0.7
45–54	0.5	0.5	0.6	0.7	0.7	0.7	0.6	0.6	0.6	0.6	0.6	0.7	0.5	0.8
55–64	0.6	0.5	0.8	0.7	0.9	0.7	0.8	0.7	0.8	0.8	0.8	0.8	0.6	0.6
65–74	0.7	0.5	0.9	0.7	0.9	0.7	0.9	0.7	0.9	0.7	1.0	0.8	0.4	0.5
75–84	0.4	0.3	0.5	0.3	0.5	0.4	0.4	0.4	0.4	0.3	0.4	0.3	0.2	0.2
85–94	0.0	0.1	0.1	0.1	0.1	0.1	0.1	0.1	0.1	0.1	0.1	0.1	0.1	0.1
≥ 95	0.0	0.0	0.0	0.0	0.0	0.0	0.0	0.0	0.0	0.0	0.0	0.0	0.0	0.0
Total	5.3	6.3	7.0	8.6	5.7	6.6	5.5	6.6	5.2	6.2	5.4	6.0	3.9	5.3
	5.80		7.83		6.18		6.04		5.68		5.72		4.61	

*Note*. *M* men, *W* women

For SAS registered in the NHSO system, the highest age-standardized incidence rate was 7.83 per 100,000 people in 2014 (8.6 in women and 7.0 in men). Subsequently, the rate decreased to approximately 38.4% for women and 44.3% for men at the end of this period (2019). Additionally, the highest rate of SAs occurred in young people aged 15–24 years of both sexes throughout this period (Table 2B).

### Trends in the annual incidence rates of successful and attempted suicides in 2013–2019

**Fig. 1:**
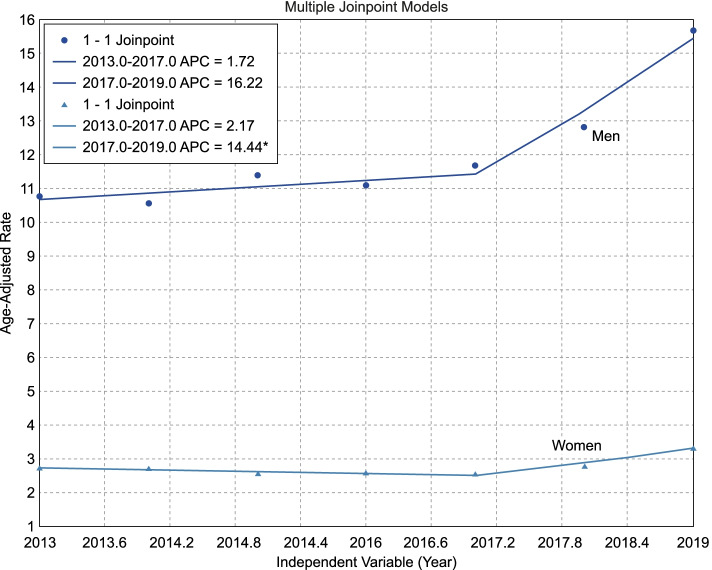
Increasing trend of age-adjusted annual suicide incidence rates by sex

**Fig. 2:**
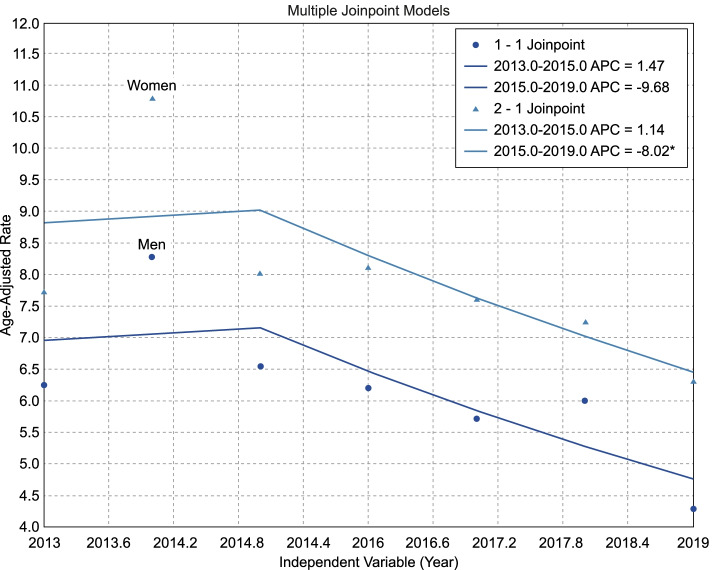
Decreasing trend in age-adjusted annual incidence rates among attempted suicide rates by sex

Table 3 and Figs. 1 and 2 outline trends in annual incidence rates of age-standardized successful and attempted suicides in Thai men and women. Our join-point regression analysis revealed significantly increasing trends of annual suicide incidence rates for both sexes, with an average change of 6.3% (95% confidence interval [CI]: 3.0, 9.8; *p* < .001) and 3.1% (95% CI: 1.2, 5.0; *p* = .001), respectively, from 2013 to 2019. Notably, significantly increasing trends were observed in 2017 in both sexes when the suicide incidence rate increased by 16.2 and 14.4% per year thereafter. Conversely, from 2013–to 2019, a significantly decreasing trend in the annual SAS incidence rates was observed among women (average incidence change: -5.1, 95% CI: − 7.9, − 2.1 per year; p = .001). A marked change was observed in 2015, with an average annual decrease in incidence rates to − 8.0% (95% CI: − 13.1, − 2.5) per year from 2015 to 2019. Among men, the trend’s pattern resembled that of women, but with an overall non-significantly decreasing trend observed from 2013–to 2019, and a change occurring in 2015.

**Table 3:** Join-point regression analysis of the age-standardized annual incidence of attempted and successful suicides

Trends in the annual incidence rate of age-standardized successful suicides					
Sex	Period	Year of change	APC (95%CI)	AAPC (95%CI)	*p*
Men	2013–2017	2017	1.7 (− 4.3, 8.1)		.352
	2017–2019		16.2 (−1.8, 37.6)		.062
	2013–2019			6.3 (3.0, 9.8)	< .001
Women	2013–2017	2017	−2.2 (− 5.5, 1.3)		.114
	2017–2019		14.4 (3.4, 26.7)		.029
	2013–2019			3.1 (1.2, 5)	.001
Trends in the annual incidence rate of age-standardized suicide attempts					
Men	2013–2015	2015	1.5 (−47.6, 96.3)		.933
	2015–2019		−9.7 (− 28.9, 14.7)		.208
	2013–2019			−6.1 (−17, 6.3)	.318
Women	2013–2019	2015	1.1 (−14.1, 19.1)		.794
	2015–2019		−8.0 (−13.1, −2.5)		.025
	2013–2019			−5.1 (−7.9, − 2.1)	.001

*Note*. *APC* annual percent change, *CI* confidence interval, *AAPC* annual average percent change; *p*-values were gained from t-tests

### Predictors of attempted and successful suicides

A multivariable logistic regression analysis using the stepwise backward elimination method showed that variables significantly associated with SAS were female sex; being aged 15–54 years; and having any mental-, alcohol-, or substance-related disorder (Table 4). After adjusting for other variables in the model, women were 1.79 times (95% CI: 1.84, 1.74) more likely to have attempted suicide than men. The odds of having a prior SAS were highest among those aged 15–24 years compared to those aged 10–14 years, then decreasing in higher age groups, with the lowest odds being among those aged 45–54 years. Having a mental disorder was the strongest predictor of SAS (adjusted OR: 18.37, 95% CI: 18.37, 19.00), whereas substance- and alcohol-related disorders increased the likelihood of SAS by 3.66 and 5.06 times, respectively (Table 4).

**Table 4:** Predictors of suicide attempts using multivariable logistic regression analyses

Predictors of SAS	Crude OR (95% CI)	Adjusted OR (95% CI)	*P*-value (Wald’s test)
Female gender	1.5 (1.46,1.54)	1.78 (1.73,1.83)	< .001
Age 10-14 yr -ref			
15–24 yr	2.65 (2.54,2.77)	2.49 (2.37,2.61)	< .001
25–34 yr	2.32 (2.2,2.45)	1.69 (1.59,1.8)	< .001
35-44 yr	1.38 (1.31,1.45)	0.92 (0.87,0.97)	0.005
45–54 yr	0.69 (0.65,0.73)	0.55 (0.52,0.59)	< 0.001
55-64 yr	0.36 (0.34,0.38)	0.35 (0.33,0.36)	< 0.001
65-74 yr	0.27 (0.26,0.28)	0.27 (0.25,0.28)	< 0.001
75-84 yr	0.25 (0.23,0.26)	0.23 (0.22,0.25)	< 0.001
85-94 yr	0.21 (0.18,0.23)	0.17 (0.15,0.19)	< 0.001
95+	0.17 (0.09,0.3)	0.13 (0.07,0.24)	0.001
Disability	0.99 (0.88,1.12)	0.79 (0.68,0.91)	0.002
Mental disorders.	22.57 (21.88,23.27)	17.92 (17.32,18.54)	< 0.001
Alcohol-related dis.	5.25 (4.84,5.7)	4.83 (4.37,5.34)	< 0.001
Substance-related dis	11.11 (9.47,13.03)	3.47 (2.84,4.25)	< 0.001
No psychiatric treat	14.87 (13.35,16.56)	1.59 (1.39,1.82)	< 0.001

*SAS* suicide attempts, *CI* confidence interval, *OR* odds ratio

*N*=513, 353

Enter model with AIC156635.7193; Log-likelihood = -78301.8597 No. of observations= 502826

We conducted a subgroup analysis among those who attempted suicide in the NHSO dataset to identify predictors of successful suicides during or after hospitalization following their first SAS. We found that men had an approximately 18% increased risk of dying from their first SAS compared to women. The odds of dying by suicide increased markedly by age group: those aged 65–74, 75–84, 85–94, and 95 years or older had the highest odds of dying via suicide (adjusted OR = 31.91, 56.75, 73.95, and 122.65, respectively). Furthermore, those who did not receive psychiatric treatment during their hospitalization were at increased risk of dying by suicide either during or after hospitalization. However, surprisingly, having either a mental- or alcohol-related disorder was associated with decreased risk of dying by suicide in this subgroup (Table 5).

**Table 5:** Predictors of deaths among suicide attempters using logistic regression analyses

Predictors of deaths	Crude OR (95% CI)	Adjusted OR (95% CI)	P-value (Wald’s test)
Men	2.77 (2.59, 2.95)	2.14 (1.99, 2.30)	< 0.001
Age 10–14 yr -ref			
15–24 yr	2.24 (1.68,2.99)	1.86 (1.39,2.48)	< 0.001
25-34 yr	7.65 (5.75,10.2)	5.56 (4.16,7.42)	< 0.001
35-44 yr	11.98 (9.08,15.82)	9.85 (7.44,13.04)	< 0.001
45-54 yr	16.81 (12.76,22.14)	14.05 (10.64,18.55)	< 0.001
55-64 yr	26.41 (20.17,34.59)	20.96 (15.97,27.51)	< 0.001
65-74 yr	41.78 (31.9,54.72)	32.46 (24.74,42.6)	< 0.001
75-84 yr	74 (55.97,97.84)	57.77 (43.61,76.54)	< 0.00100
85-94 yr	100.01 (70.01,142.87)	74.81 (52.12,107.36)	< 0.001
95+ yr	157.24 (40.68,607.78)	124.03 (31.39,490.07)	< 0.001
High lethal method	2.16 (1.95,2.4)	1.72 (1.53,1.94)	< 0.001
Disability	0.8 (0.57,1.12)	0.66 (0.46,0.95)	0.026
Mental disorder(s)	0.6 (0.56,0.64)	0.62 (0.58,0.67)	< 0.001
Alcohol-related disorder	1.15 (0.96,1.37)	0.64 (0.53,0.77)	< 0.001

Stepwise backward model with AIC value = 19455.0717, Log-likelihood = -9711.5359

No. of observations = 26135

## Discussion

This study shows trends in successful and attempted suicides among the general Thai population from 2013 to 2019. There was a significantly increasing trend in successful suicide during the 7 years, from 6.12 in 2013 to 8.95 per 100,000 people in 2019. The increase was notable among men, whose annual incidence rate increased from 9.8 in 2013 to 14.8 per 100,000 in 2019 (around 6.3% per year). The suicide trend in women also increased to a smaller extent, from 2.6% in 2013 to 3.4 per 100,000 in 2019 (3.1% per year). A spike occurred in 2017–2019; annual suicide incidence rates increased by an average of 16.2 and 14.4% per year among men and women, respectively. These findings resemble those reported in prior studies outlining the higher incidence of suicide in men than women. One explanation of the higher successful suicide rate in men is their tendency to use high-lethality methods, such as hanging, guns, and jumping from tall structures [23–29]. Men have higher rates of alcohol and substance use, as well as impulsivity, than do women [30].

The increasing trend of suicides from 2017 to 2019 is attributable to the occurrence of many critical/largescale events. Multiple factors could be related to the increasing suicide rate. For instance, during the first 3 months (November 2016 - January 2017) on October 13, 2016, King Bhumibol Adulyadej died at Siriraj Hospital in Bangkok after a 70-year reign. A year of  mourning was declared the following day. Thai people dramatically in their daily lives and worldviews. People wore black dressing to work. Royal portraits were displayed in front of business buildings, whether they were single-story buildings or skyscrapers. These affected the atmosphere of national grievance [31].  Additionally, in January 2017, flooding diaster occurred in the southern region, with a death toll of at least 95 people and affecting an additional 1.8 million people [32].

Although economic conditions in Thailand improved in 2017 [33], in 2019, Thailand was affected by the global trade slowdown owing to intensifying trade tensions between the US and China. Subsequently, exports and manufacturing production contracted. Business sentiment and investments were negatively affected throughout the year by international trade protectionism measures, Brexit, and various geopolitical conditions. Thai economic growth slowed from 4.2% in 2018 to 2.4% in 2019. Furthermore, between 2015 and 2018, the poverty rate in Thailand increased from 7.2 to 9.8%. The number of people living in poverty rose from 4.85 million to more than 6.7 million. However, from 2018 to 2019, the poverty rate did drop to 6.2% [34].

Additionally, the annual incidence rate of SAS was highest in 2014 (7.83 per 100,000 people) among both women (8.6 per 100,000) and men (7.0 per 100,000). The consistently higher rates of SAs in women identified here and elsewhere may reflect the higher risk of depression of this sex [35–37]. However, from 2013–to 2019, a significantly decreasing trend of SAS was observed among women (average incidence change: -5.1, 95% CI: − 7.9, − 2.1 per year; *p* = .001), peaking in 2015. Among men, the trend was similar, but with a non-significantly decreasing trend in 2013–2019 and a change point occurring in 2015. Our results differ from those of Xiao et al. (2021), who reported that the prevalence of SAS increased among adolescents; however, no significant trends were observed from 1991 through 2019. We found a substantial decrease in SAS compared to the overall increasing suicide rate. This may reflect that most young people who engage in self-injury do not seek medical treatment [38]; thus, the SAS estimates derived from hospital admissions likely underrepresent the true extent of self-injury among young people. First-attempters were more likely to use highly lethal methods, less likely to have known mental health problems or disclose their intent to others, and more likely to successfully suicide within the context of a specific stressful circumstance [39].

Predictors of SAS were found significantly among women; young individuals (15–24 years); those with mental health-, alcohol- or substance-related disorder(s); and those who had not received psychiatric treatment, with odds ratios reported at 1.32, 20.51, 5.23, 4.69, and 1.81, respectively. This finding is consistent with reports from Western countries [40–42] and East Asia [42–44]. Furthermore, a study of the NHSO dataset revealed 136,265 fatal cases (26.5% death rate) out of the recorded 513,353. Out of 26,178 cases, 4853 were fatal SAS. Thus, the death rate from attempted suicide was 18.5%. The risk of death due to SAs was significantly higher among older men, those who attempted suicide using highly lethal methods, and those who did not receive psychiatric treatment. These findings are consistent with those of previous studies [42, 45]. Additionally, these results indirectly indicate that psychiatric consultations are beneficial for those who attempt suicide, similar to the findings of Suokas and Lönnqvist [13]. However, in contrast to previous literature, our study did not find that mental and alcohol-related disorders predicted death among individuals who attempted suicide. A possible explanation could be that these patients were often treated at emergency clinics and underwent consultation for psychiatric treatment.

Our implications highlight sex-related gaps in public health owing to an identified higher incidence of suicide among men, and a higher incidence of SAS in women adolescents, emphasizing the need to consider sex-sensitive issues in individual stress-diathesis risks and larger societal-contextual factors. Predictors of SAS in our study were female sex; being an adolescent or young adult; and having any mental, alcohol-, or substance-related disorder(s). However, prior studies have identified other factors, including adverse childhood experiences, domestic violence, interpersonal hardships, and socioeconomic disparities; these should be included in risk assessments. Our findings underscore the need for more funding support, policy advocacy, and suicide surveillance systems to develop more comprehensive and culturally appropriate prevention programs targeting different risk categories and methods across at-risk groups. Prevention strategies, including health promotion campaigns to increase public awareness of suicidal behaviors and warning signs, encouraging people to seek help from mental health professionals, risk screening and assessment strategies, resilience programs, coping and problem-solving skills training, and instituting policies that strengthen people’s financial stability would all help to improve the public’s suicide literacy, thereby lowering the overall suicide rates. Moreover, emphasis should be placed on improvement of accurate reporting the causes of death, especially suicide through training programs for medical students, residency training, other healthcare providers and local civil registrars. Public awareness of suicide prevention should be advocated, destigmatized for persons with SAS and increased accessibility to mental heath services.   

### Study strengths and limitations

Our use of a large sample of nationally representative, recent mortality data is a strength. It offers a unique opportunity to examine trends in successful and attempted suicides among the Thai population. Our relatively large sample is representative of the overall population’s suicidal behaviors. Thus, our findings are both valid and generalizable. Our study has some limitations regarding the availability and reliability of routinely reported mortality data. Aside from general reluctance to report deaths by suicide owing to societal stigma, inadequacies exist in death registration practices, with incomplete or erroneous entries for the cause of death occurring existing in official records. Certification is generally based on patient clinical symptoms rather than autopsy results, even in medically attended deaths. Although death certificate completion guidelines exist, these frequently are not followed [46–48]. Furthermore, the profile of people who attempt suicide was derived from hospital-based data recorded from 2013–to 2019, covering different periods; however, suicide patterns, environments, and beliefs could be different herein. Finally, our study did not examine other potentially influential factors, including adverse childhood experiences, domestic violence, personality disorders, major life events, stressful life situations, financial difficulties, and interpersonal hardships.

## Conclusion

The annual age-standardized suicide rate in Thailand increased from 6.12 per 100,000 people in 2013 to 8.95 per 100,000 in 2019, was higher among people of working age (25–64 years) and adolescent men, and showed an increasing trend over this period and a change in 2017. In contrast, trends of annual age-standardized SA rates decreased 7.83 per 100,000 people in 2014 to 4.61 in 2019 and were significantly lower for women, with a notable change in 2015. The identified predictors of SAs included female sex, being younger, having a disability, and having a mental/alcohol/substance-related disorder(s). Predictors of death by suicide included male sex, being older, having a mental disorder(s), having an alcohol-related disorder, having previously attempted suicide, and receiving no psychiatric treatment. These findings highlight the need for more reliable data from national surveillance systems to understand suicidal behaviors, risks, and protective factors to establish culturally appropriate prevention strategies in Thailand.

## Data Availability

The data that support the findings of this study are available from the Bureau of Policy and Strategy of the Ministry of Public Health at [http://ghdx.healthdata.org/organizations/bureau-policy-and-strategy-ministry-public-health-thailand], and the NHSO [https://www.nhso.go.th/].

## Abbreviations

NHSO: National Health Security Office
SAS: Suicide attempts
